# Activation of PDGFr-β Signaling Pathway after Imatinib and Radioimmunotherapy Treatment in Experimental Pancreatic Cancer

**DOI:** 10.3390/cancers3022501

**Published:** 2011-05-25

**Authors:** Michio Abe, Zbigniew P. Kortylewicz, Charles A. Enke, Elizabeth Mack, Janina Baranowska-Kortylewicz

**Affiliations:** 1 Minamata City Hospital and Medical Center, Minamata City, Kumamoto 867, Japan; E-Mail: PFG02651@nifty.ne.jp; 2 Department of Radiation Oncology, J. Bruce Henriksen Cancer Research Laboratories, University of Nebraska Medical Center, Omaha, NE 68198, USA; E-Mails: zkortylewicz@unmc.edu (Z.P.K.); cenke@unmc.edu (C.A.E.); elizabeth.mack@unmc.edu (E.M.)

**Keywords:** pancreatic cancer, PDGFr-β, PDGF-B, imatinib, radioimmunotherapy, radiation

## Abstract

Pancreatic cancer does not respond to a single-agent imatinib therapy. Consequently, multimodality treatments are contemplated. Published data indicate that in colorectal cancer, imatinib and radioimmunotherapy synergize to delay tumor growth. In pancreatic cancer, the tumor response is additive. This disparity of outcomes merited further studies because interactions between these modalities depend on the imatinib-induced reduction of the tumor interstitial fluid pressure. The examination of human and murine PDGFr-β/PDGF-B pathways in SW1990 pancreatic cancer xenografts revealed that the human branch is practically dormant in untreated tumors but the insult on the stromal component produces massive responses of human cancer cells. Inhibition of the stromal PDGFr-β with imatinib activates human PDGFr-β/PDGF-B signaling loop, silent in untreated xenografts, via an apparent paracrine rescue pathway. Responses are treatment-and time-dependent. Soon after treatment, levels of human PDGFr-β, compared to untreated tumors, are 3.4×, 12.4×, and 5.7× higher in imatinib-, radioimmunotherapy + imatinib-, and radioimmunotherapy-treated tumors, respectively. A continuous 14-day irradiation of imatinib-treated xenografts reduces levels of PDGFr-β and phosphorylated PDGFr-β by 5.3× and 4×, compared to earlier times. Human PDGF-B is upregulated suggesting that the survival signaling via the autocrine pathway is also triggered after stromal injury. These findings indicate that therapies targeting pancreatic cancer stromal components may have unintended mitogenic effects and that these effects can be reversed when imatinib is used in conjunction with radioimmunotherapy.

## 1. Introduction

Pancreatic cancer cells have a broad array of cellular defects promoting their uncontrolled growth. Of these, the most extensively studied is the transduction of extracellular growth stimulating signals to the cell nucleus by the HER family of kinases [1-5]. The introduction of clinically important platelet-derived growth factor (PDGF) inhibitors such as imatinib mesylate (previously STI571, Glivec® or Gleevec® in the USA) shifted some of this attention to the PDGF signaling pathways for therapeutic purposes [6-11]. Platelet-derived growth factor receptors β (PDGFr-β) are involved in the autocrine and paracrine stimulation of solid tumor growth [11]. They also regulate cell proliferation and migration, and activate endothelial cells. Blocking PDGFr-β pathways retards angiogenesis, vascular maturation, and cell proliferation, leading to tumor regression [12,13]. In rat gliomas and in pancreatic islet tumors, imatinib and other PDGFr-β inhibitors force tumor blood vessel into regression through the pericyte-associated pathways involved in the vascular stabilization and maturation [14,15]. A great deal of evidence suggests that normal cells from the stroma, including endothelial cells, fibroblasts, immune and inflammatory cells are recruited into pancreatic tumors. These various cell types of mesenchymal origin express PDGFr-β and use this signaling loop in the recruitment of tumor stroma fibroblasts, and stimulation of tumor angiogenesis. Paracrine PDGFr-β stimulation enhances solid tumor growth through the recruitment of vascularized stroma [12-15].

The expression of c-Kit [16] and PDGFr-β [11,17] in a large number of pancreatic cancer biopsies was regarded as an encouraging sign and several clinical protocols involving imatinib were initiated. Currently, the efficacy of imatinib has been tested in clinical trials in treatment of at least a dozen solid tumors [18-23]. Despite unprecedented successes of imatinib in chronic myelogenous leukemia and preclinical *in vivo* studies in various mouse models suggesting that imatinib may be useful in the treatment of solid cancer [24-27], most solid tumors do not respond to imatinib therapy [7,10,19-23]. Recently published reports on the clinical effects of imatinib in a small group of patients with unresectable pancreatic cancer showed no objective responses [7,10]. The expression of c-Kit and PDGFr-β was confirmed immunohistochemically in the biopsy samples in this group of patients. The lack of clinical efficacy in these and other clinical trials is not fully understood and often attributed to the insufficient PDGFr-β expression.

Studies described below show that the pretreatment status of PDGFr-β and its ligand PDGF-B in human pancreatic cancer xenografts is radically altered during therapeutic intervention with imatinib, radioactivity or both. Unlike in the case of the LS174T colorectal cancer [28], imatinib alone produces significant regression of SW1990 pancreatic cancer xenografts [27] suggesting dissimilar or additional interactions between radioimmunotherapy (RIT), imatinib, and pancreatic cancer cells. SW1990 cells grown in vitro have low levels of functional PDGFr-β [27] and conceivably these may have been sufficient to influence tumor responses. The comprehensive evaluation, reported here, of the SW1990 tumors reveals unexpected trends in the PDGFr-β expression and activation.

## 2. Results and Discussion

### 2.1. PDGFr-β Expression in SW1990 Human Pancreatic Cancer Xenografts

**Figure 1.**
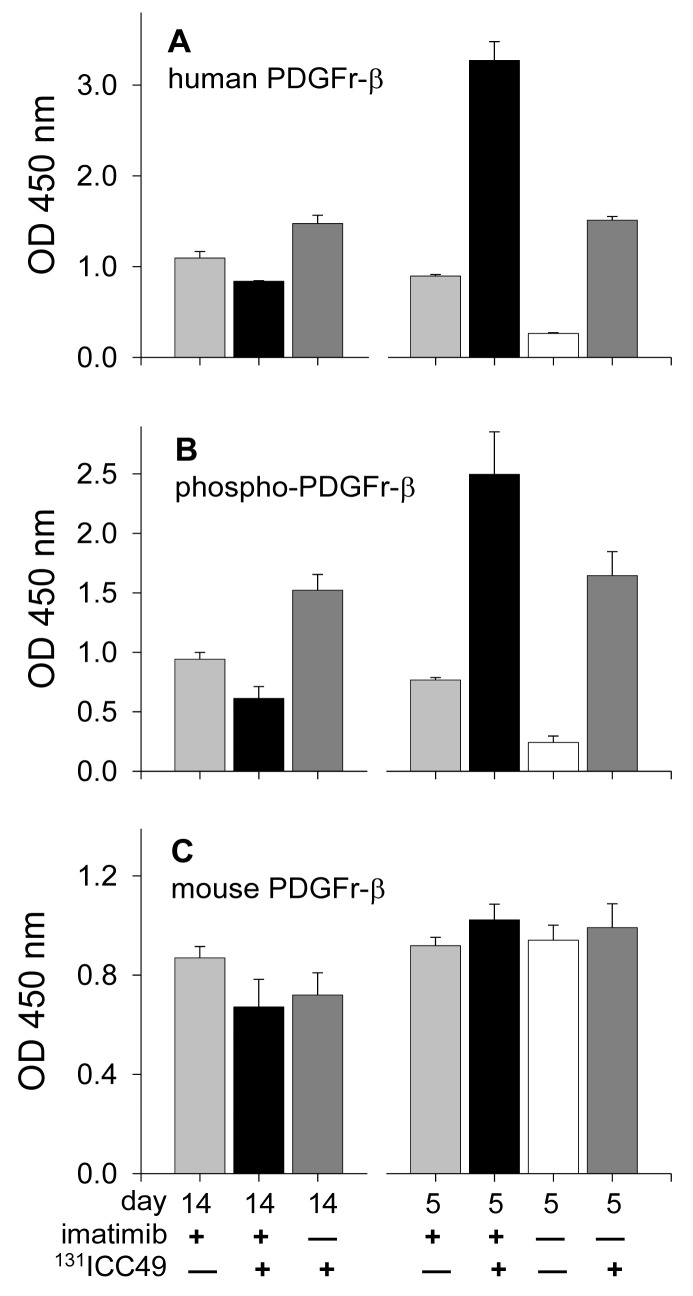
Expression of various forms of PDGFr-β in lysates prepared from SW1990 human pancreatic adenocarcinoma xenografts extirpated 5 d and 14 d after various treatments and measured using commercial ELISA kits. White bars represent the expression of PDGFr-β in tumors from control mice. Note different scales of the y-axes.

ELISA analyses offer compelling evidence that levels of PDGFr-β strongly depend on the treatment employed, time elapsed after this treatment, and the source of the receptor, *i.e.*, whether of the human origin derived from tumor cells or of the mouse origin derived from mesenchymal cells of the host (Figure 1). Tumors extirpated from the control mice that received sham IP injections of PBS showed only low levels of human PDGFr-β (Figure 1A) and correspondingly low levels of phosphorylated PDGFr-β (Figure 1B). The magnitude of mouse PDGFr-β expression was nearly identical in all tumor samples recovered five days after the treatment irrespective of what this treatment was (Figure 1C). On the other hand, levels of human PDGFr-β expression greatly depended on the treatment. In tumors recovered five days after treatment, human PDGFr-β levels were 3.4×, 12.4×, and 5.7× higher in the imatinib-treated (light gray bar), ^131^ICC49 + imatinib-treated (black bar), and ^131^ICC49-treated mice (dark gray bar), respectively, as compared to PBS-treated control mice (white bar). The receptor phosphorylation followed along the same lines with the treated tumors showing on average 3.1×, 10.3×, and 6.6× higher levels of phosphorylation than tumors from PBS-treated mice demonstrating that the primary response of the tumor tissue to the insult by imatinib or by radiation from ^131^ICC49 is the increased phosphorylation of the human PDGFr-β receptor. PDGFr-β associated with the stroma, *i.e.*, receptors of the mouse origin, do not seem to respond to any of applied treatments in a major way. No significant deviations in levels of mouse PDGFr-β in treated tumors as compared to those measured in tumors from control mice were detected (Figure 1C).

**Figure 2.**
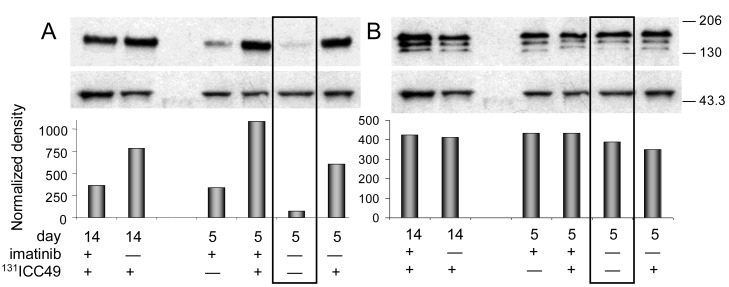
Expression of PDGFr-β in SW1990 human pancreatic SW1990 adenocarcinoma xenografts grown subcutaneously in athymic mice. **(a)** Phosphorylated PDGFr-β of human and mouse origin. Western immunoblotting analyses of phosphorylated PDGFr-β with rabbit anti-human/anti-mouse phospho-PDGFr-β (Tyr751) polyclonal Ab3161 antibodies. The lower panel is the same membrane probed with rabbit anti-β-actin mAb 13E5. The bar graph represents protein band intensity normalized to the protein load as measured by the intensity of β-actin band using ImageJ. **(b)** Total PDGFr-β. Western immunoblotting analyses with rabbit anti-human/anti-mouse PDGFr-β polyclonal Ab3162 to determine the total expression of PDGFr-β in response to various treatments. The lower panel is the same membrane probed with rabbit anti-β-actin mAb 13E5 to determine total protein load per lane. The bar graph represents protein band intensity normalized using ImageJ to the protein load as measured by the intensity of β-actin band. Immunoblots for β-actin are identical in A and B because the same membrane was re-probed. Data for xenografts from control mice sham-treated with PBS are framed by the black rectangle.

**Figure 3.**
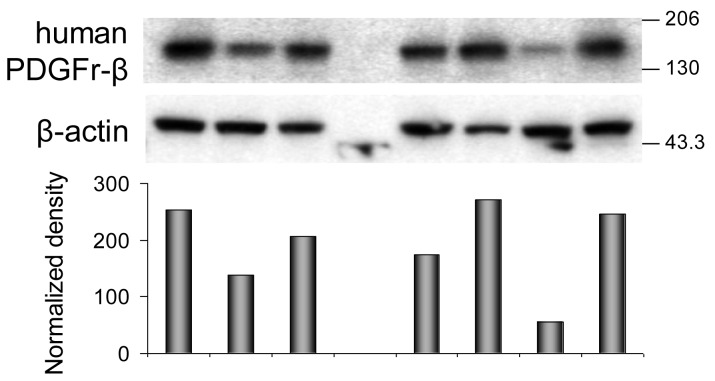
Activation of human PDGFr-β signaling pathway in response to imatininb and radiation injury. Expression of human PDGFr-β. Western immunoblotting analyses with mouse monoclonal anti-human PDGFr-β monoclonal antibodies ab10847 to determine expression of human PDGFr-β in response to various treatments. The bands were detected with ImmunoPure goat anti-mouse IgG (H + L), peroxidase conjugated antibodies 31430. The lower panel is the same membrane probed with rabbit anti-β-actin mAb 13E5. The bar graph represents protein band intensity normalized to the protein load as measured by the intensity of β-actin band using ImageJ.

Western immunoblotting analyses confirmed the ELISA findings (Figure 2 and Figure 3A). Phosphorylated PDGFr-β (Figure 2A) in PBS-treated tumors is barely detectable. All tumor samples collected on day five after treatment consistently follow the trends detected with the ELISA. Differences calculated from the protein band intensity are slightly more pronounced (Figure 2A, bar graph) compared to the results of ELISA probably because the threshold of the Western blot sensitivity is well beyond the sensitivity of the ELISA method. The specificity of reagents available for the immunoblotting of mouse PDGFr-β is not up to par with the ELISA's antibodies. For this reason, the Western blot shown in Figure 2B illustrates the total PDGFr-β expression, *i.e.*, mouse and human PDGFr-β, as well as phosphorylated PDGFr-β of both origins. When the protein band intensity is calculated to account for the slight variations in the total protein applied (Figure 2B, bar graph), these results are practically indistinguishable from the ELISA data, *i.e.*, the treatment has little or no effect on the expression of mouse PDGFr-β five days after treatment. In contrast, the expression of human PDGFr-β as detected by the immunoblotting is strongly dependent on the treatment and is perfectly comparable with the ELISA results (Figure 3A). The inclusion of either imatinib, ^131^ICC49, or both, in the SW1990 treatment stimulates human PDGFr-β expression well beyond the basal levels.

The ELISA examination of samples recovered 14 d after treatment revealed further significant alterations in the PDGFr-β expression. Phosphorylation of PDGFr-β was suppressed in ^131^ICC49 + imatinib treated samples and was as much as four times lower compared to levels measured on day five after treatment (Figure 1B). Imatinib-treated tumors showed a slight increase in the phosphorylation of PDGFr-β, specifically OD at 450 nm for 5-d tumor samples was 0.77 ± 0.02 compared to 0.94 ± 0.06 in 14-d samples (P = 0.0075). ^131^ICC49-treated tumors had levels of phosphorylated PDGFr-β virtually unchanged, OD 450 nm were 1.64 ± 0.20 and 1.52 ± 0.13 five and 14 d after treatment, respectively (P = 0.429). Mouse PDGFr-β was suppressed in the ^131^ICC49 + imatinib-and ^131^ICC49-treated tumors by ∼30% (P = 0.009), whereas imatinib-treated tumors showed only a minor decrease, ∼5%, in mouse PDGFr-β levels (Figure 1C). The expression of human PDGFr-β was influenced by the treatment much like the PDGFr-β phosphorylation levels. Tumors, which were treated with ^131^ICC49 + imatinib, showed 5.3× lower levels of human PDGFr-β in tumors collected 14 d after treatment as compared to the 5-d tumor samples (P < 0.0001). The imatinib treatment at 5 d had more effect than at 14 d. The level of human PDGFr-β was ∼18% higher in the 14 d sample (Figure 1A). ^131^ICC49 had no time-dependent effect on the expression of human PDGFr-β, OD 450 nm were measured at 1.51 ± 0.04 and 1.47 ± 0.09 for the 5 d and 14 d tumors, respectively (P = 0.564). The corresponding Western immunoblotting data is shown in Figure 2 and Figure 3A.

### 2.2. PDGF-B Expression in SW1990 Human Pancreatic Cancer Xenografts

**Figure 4.**
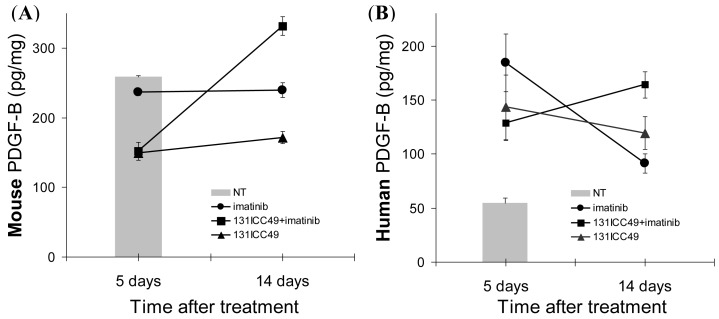
Time-and treatment-dependent changes in the expression of mouse PDGF-B (**A**) and human PDGF-B (**B**).

The changes in the PDGFr-β expression were associated with significant alterations in tumor-associated PDGF-B (Figure 4). Human PDGF-B in tumors from control mice treated only with PBS was measured at 54.7 ± 4.6 pg/mg total protein. Mouse PDGF-B in these control tumors was 258.4 ± 1.98 pg/mg total protein. Five d after the treatment, the expression of mouse PDGF-B fell in all treatment groups to 236.4 ± 4.6 pg/mg, 152.3 ± 12.8 pg/mg, and 149.6 ± 2.3 pg/mg in imatinib (*P* = *0.048*), ^131^ICC49 + imatinib- (*P* = *0.015*), and ^131^ICC49-treated (*P* = *0.0008*) tumors, respectively. In contrast, human PDGF-B was elevated from ∼55 pg/mg protein before treatment to 184.4 ± 26.9, 128.4 ± 15.8, and 143.3 ± 30.0 pg/mg in the corresponding day 5 tumor samples from mice treated with imatinib (*P* = *0.034*), ^131^ICC49 + imatinib (*P* = *0.046*), and ^131^ICC49 (*P* = *0.107*). After 14 days, mouse PDGF-B in imatinib-treated tumors persisted at essentially unchanged levels, 239.7 ± 10.4 pg/mg (*P* = *0.219*). ^131^ICC49-treated tumors showed mouse PDGF-B of 332.0 ± 13.6 pg/mg at 14 d, an increase of ∼74 pg/mg (*P* = *0.010*). In ^131^ICC49 + imatinib-treated tumors, the amount of mouse PDGF-B increased by 20 pg/mg protein and was registered at 171.6 ± 8.6 pg/mg (*P* = *0.033*). The amount of human PDGF-B in imatinib-treated tumors measured 14 d after treatment was still higher than levels measured in untreated tumors but it declined to 91.4 ± 8.83 pg/mg (*P* = *0.065*). ^131^ICC49 treatment resulted in 119.3 ± 15.0 pg/mg (*P* = *0.054* approximately 120% of PDGF-B levels measured on day 5. The combined treatment in ^131^ICC49, + imatinib promoted the raise of human PDGF-B levels to 164.0 ± 12.6 pg/mg (*P* = *0.014*), ∼36 pg/mg higher than in 5 d samples, and nearly 3× higher than in tumors from PBS-treated control mice (Figure 4B).

### 2.3. Immunohistochemistry of SW1990 Human Pancreatic Cancer Xenografts

**Figure 5.**
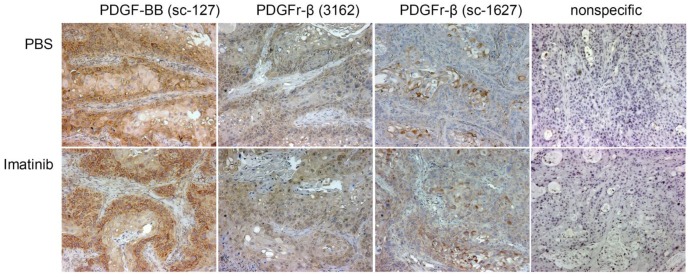
Immunohistochemistry of PDGF-B and PDGFr-β in xenografts extirpated from PBS-and imatinib-treated mice. Original magnification 10×, counterstained with hematoxylin.

The PDGFr-β and PDGF-B expression was also evaluated using immunohistochemistry (Figure 5). Xenografts are heterogeneous and for this reason, any quantitative assessment of the relative expression of the growth factor and its cognate receptor is not possible. Microscopically, xenografts show atypical and irregular glandular structures and solid tumor masses supported by bands of fibrovascular stromal component (Figure S1 in the supplementary material).

### 2.4. Discussion

The goal to improve diagnosis and therapy of pancreatic cancer focused research efforts on molecular abnormalities present in this disease with much attention paid to the role of growth factors and their allied receptors [1-5,17,31-34]. Growth factors can wield a wide range of effects via autocrine and paracrine signaling loops. It is well understood that tumor can regulate the development of its stromal component through the anomalous induction of growth factor receptors and the production of their ligands. It is also understood that there is a degree of reciprocity involved in this communication, *i.e.*, tumor stroma can equally influence tumor cells. This type of a continuous mutual regulation of interactions between tumor cells and their environment are held responsible for pancreatic cancer development and progression by promoting survival, migration, invasion, and angiogenesis. For this reason, increasing number of clinical trials in pancreatic cancer include drugs that target the stroma. One way to target stroma is through the inhibition of PDGFr-β in hopes of damaging the stromal compartment to such an extent that it is incapable of providing any support to tumor cells.

The reduction of tumor's interstitial fluid pressure in response to imatinib is one of the hypothesized stromal effects of imatinib and the source of the improved tumor responses to imatinib-augmented radiotherapy [27,28]. Immunohistochemistry confirmed that the most likely primary target was the stromal PDGFr-β, given that all detectable PDGFr-β was localized exclusively in the mesenchymal cells [28]. Immunohistochemistry of PDGFr-β in SW1990 model was ambiguous but the receptor also appeared to be confined primarily to the stromal component. In vitro studies showed that SW1990 tumor cells have low but functional levels of PDGFr-β responsive to imatinib [27]. In view of the results in the colorectal cancer model [28], the significant growth delay of SW1990 xenografts in mice treated only with imatinib was unforeseen. Moreover, most of the successful single modality therapies with imatinib in various cancer models required 5 - 8 weeks of twice a day dosing of imatinib [24,26,30]. In studies reported here, the growth delay was already apparent after three days of dosing with imatinib at 100 mg/kg BID [27].

Immunohistochemical analyses of human pancreatic cancer biopsies and xenografts extirpated from mice show expression of PDGFr-β and PDGF-B mainly on cells of the tumor stroma. Treatment with imatinib inhibits PDGFr-β phosphorylation but reportedly, the overall expression of this receptor is not altered [17,24,34]. In contrast, our results show remarkable treatment-and time-dependent variations in the expression of cancer cell-associated PDGFr-β and its cognate ligand PDGF-B. These changes were corroborated by two techniques, ELISA and immunoblotting. The majority of the phosphorylated receptor derives from the human cancer cells. What accounts for this remarkable response of the tumor cell must be further investigated. Meanwhile, we hypothesize that the initial injury of the tumor stroma by PDGFr-β inhibition with imatinib or by irradiation with ^131^ICC49 triggers an immediate and massive feedback from tumor cells. The response seems to be autocrine and paracrine. Tumor levels of human PDGF-B increase twofold in response to all single and multimodality treatments after five days compared to tumors from PBS-treated controls. Tumors analyzed 14 days after treatment also show much increased production of human PDGF-B. By comparison, these changes are accompanied by a substantial suppression in the production of mouse PDGF-B in all but ^131^ICC49 + imatinib tumors collected on day 14 after treatment. Is this indicative of the tumor cell response to the damaged stroma? Possibly. A change in the expression of some growth factors is often associated with the analogous change in the expression of the cognate receptors.

Previous studies implicated the autocrine loop caused by the high expression of PDGF-B and PDGFr-β in the proliferation of some glioblastomas [35,36]. Here, we demonstrate that in untreated human pancreatic cancer cells PDGFr-β/PDGF-B signaling mechanism appears to be dormant in the absence of the stromal injury. The basal levels of human PDGFr-β and human PDGF-B are low. The primary target for imatinib is the stromal receptor. Likewise, ^131^ICC49 first targets the extracellular antigen TAG-72. Many tumors show diffuse cytoplasmic TAG-72 but there are no reports from the *in vivo* studies on the intracellular localization of B72.3 or CC49, two antibodies most often used to target this antigen [37-39].

There is a striking similarity of tumor cell responses to the stromal insult with radiation or PDGFr-β inhibition. In both cases, human PDGFr-β expression increases and so does its phosphorylation -the first step in the signaling pathway. A parallel escalation in the production of human PDGF-B follows. PDGF-B is a mitogenic polypeptide involved in cellular proliferation and tissue repair. The increase in its levels may be indicative of a paracrine rescue pathway whereby cancer cells commence production of a trophic growth factor when stromal component originating from a host, in this case a mouse, partially loses its ability to make this factor. Mouse PDGF-B levels in tumors treated with ^131^ICC49 and ^131^ICC49 + imatinib are ∼50% of the levels detected in untreated samples. The increased expression of human PDGFr-β also suggests awakening of the autocrine signaling loop. Tumor cell responses measured at 14 d after stromal injury are not as notable, still a human branch of the PDGFr-β/PDGF-B signaling pathway, practically silent before treatment, is a great deal more active. This loop appears to be significantly disrupted in tumors treated with a combination of radioactivity and imatinib. The expression of human PDGFr-β expression and its phosphorylation are significantly suppressed in ^131^ICC49 + imatinib-treated tumors compared to single modality treatments. Levels of mouse PDGFr-β in ^131^ICC49 + imatinib tumors are also reduced. The parallel increase of mouse PDGF-B in this group may be indicative of a reciprocal communication from the stromal compartment to encourage cancer cell growth.

## 3. Experimental Section

### 3.1. Reagents

Sodium iodide-131 was purchased from PerkinElmer Life and Analytical Sciences, Inc. (Boston, MA, USA). Imatinib was generously provided by Novartis Pharma AG (Basel, Switzerland). All radioiodinations were performed on site as described previously [27].

### 3.2. Tumor Model

The SW1990 cell line derived from spleen metastasis of a grade II human pancreatic ductal adenocarcinoma [29] was purchased from ATCC (American Type Culture Collection, Manassas, VA, USA). Cells were grown in Leibovitz's L-15 medium with 300 mg/L L-glutamine, supplemented with 10% fetal bovine serum at 37 °C without CO_2_. Female 4–6 weeks old athymic NCr-nu/nu mice (NCI-Frederick, MD, USA) were allowed to acclimate for no less than 5 days. All protocols involving animals were approved by the Institutional Animal Care and Use Committee. Mice were housed in micro-isolator cages with free access to sterilized standard rodent diet and water. SW1990 cells in 0.2 mL of serum-free medium were implanted SQ at 5 × 10^6^ cells/mouse. One week later identification transponders were implanted, also SQ. Body weights and tumor sizes were monitored twice weekly, and approximately 5–7 weeks after the cell implant, mice were randomized for various therapy studies.

### 3.3. Drug Treatment

Mice were treated with imatinib and ^131^ICC49 as described previously [27]. Briefly, a lottery was conducted to separate mice into four groups as follows: group 1: control mice receiving i.p. injections of PBS (b.i.d.) on days −2, −1, and 0; group 2: imatinib group, mice receiving imatinib as the i.p. doses of 100 mg/kg/day in PBS (b.i.d.) on days −2, −1, and 0; group 3: ^131^ICC49 group, mice receiving i.p. injections of PBS (b.i.d.) on days −2, −1, and 0; and group 4: ^131^ICC49 plus imatinib group, mice receiving imatinib as the i.p. doses of 100 mg/kg/day in PBS (b.i.d.) on days −2, −1, and 0. On day 0, mice in groups 3 and 4 received via a tail vein i.v. doses of ^131^ICC49 2 h after the last dose of imatinib. Sections of tumors harvested during necropsy were frozen in liquid N_2_, and stored at −80 °C until ready for ELISA and Western blotting analyses.

### 3.4. Lysates

Frozen tumors were minced and transferred into a volume of ice-cold lysis buffer equivalent to two tumor weights. The lysis buffer contained 20 mM Tris, pH 7.5, 150 mM NaCl, 1 mM ethylene diaminetetraacetic acid (EDTA), 1 mM ethylene glycolbis(2-aminoethyl)-N,N,N′,N′-tetraacetic acid (EGTA), 1% Triton X-100, 2.5 mM sodium pyrophosphate, 1 mM β-glycerol-phosphate, 1 mM activated Na_3_VO_4_, and 1μg/mL leupeptin, and was supplemented just before the use with phenylmethylsulfonyl fluoride (PMSF) to the final concentration of 1 mM. Minced tumor fragments were sonicated on ice using a Vibra Cell Model VC 375 ultrasonic processor (Sonics & Materials, Inc., Danbury, CT, USA) for 14 s with a 14-s break between sonications for the total of 3 minutes at a 40% duty cycle. The homogenate was transferred into the microcentrifuge tubes and centrifuged at 14,000× g for 10 min at 4 °C. Supernatants were aliquoted and stored at −80 °C after the total protein content was determined using the micro BCA protein assay kit (Pierce Biotechnology, Rockford, IL, USA).

### 3.5. PDGFr-β Sandwich ELISA

The assay was purchased from Cell Signaling Technology, Inc. (Danvers, MA, USA). All procedures were conducted according to the vendor's instructions for the detection of endogenous levels of phospho-PDGFr-β (Tyr751). To quantify the total PDGFr-β and human PDGFr-β, the general principle of the assay was applied with the appropriate modification in the selection of the primary and secondary antibodies. The capture antibody coated onto all wells and provided by the manufacturer was rabbit mAb 28E1, which binds non-phosphorylated and phosphorylated PDGFr-β proteins of human and mouse origin. Primary antibodies were as follows: mouse mAb 3166 anti-phospho-PDGFr-β (Tyr751) for detection of the total phospho-PDGFr-β; mouse mAb (ab 10847) anti-human PDGFr-β for detection of human PDGFr-β (Abcam, Cambridge, MA, USA); and rat mAb (clone APB5) anti-mouse PDGFr-β for detection of the murine PDGFr-β (eBioscience, San Diego, CA, USA). Horseradish peroxidase-linked (HRP) anti-mouse antibody (7076) provided with the assay was used as a secondary antibody for phospho-PDGFr-β and human PDGFr-β detection. Donkey HRP-linked anti-rat polyclonal Ab (R&D Systems, Inc. Minneapolis, MN, USA) was used to detect murine PDGFr-β. Stabilized tetramethylbenzidine (TMB) was used as a substrate to produce color. The absorbance was read at λ = 450 nm within 30 min after the addition of the “stop” solution provided with the kit.

### 3.6. Western Blot Analyses

Tumor lysates were prepared as described above. Protein concentration was measured and aliquots of 50 μg total proteins (25 μg for human PDGFr-β studies) from each tumor lysate were added to the non-reducing sample buffer. Protein samples were denatured at 95 °C for 5 min, cooled and loaded onto 4–20% gradient Tris-HCl SDS-polyacrylamide minigels (BioRad, Hercules, CA, USA). Proteins were transferred overnight onto a Hybond™-P 0.45-μm PVDF membrane (Amersham Biosciences, Piscataway NJ, USA) in a cold room using a constant voltage of 14 mV for 18 h. Kaleidoscope Prestained Standards (Bio-Rad Laboratories, Hercules, CA, USA) were used to monitor the efficiency of protein transfer. The membrane was incubated in 5% BSA in 10 mM Tris-HCl, pH 7.5, 100 mM NaCl, 0.1% Tween-20 overnight at 4 °C to reduce nonspecific binding. The blocked membrane was incubated in the same buffer containing primary antibodies overnight at 4°C. The membrane was first probed simultaneously with rabbit anti-human/anti-mouse phospho-PDGFr-β (Tyr751) polyclonal Ab 3161 and with rabbit anti-β-actin mAb 13E5 (both from Cell Signaling Technology, Danvers, MA, USA) to detect total phosphorylated PDGFr-β and β-actin, respectively, both at 1:5,000 dilution. The membrane was washed in PBS/0.5% Tween-20 for 30 min at rt with the buffer exchange every 5 min, then incubated in the blocking buffer containing HRP-linked goat anti-rabbit IgG secondary antibody 7074 at a 1:40,000 dilution for 1 h at rt (Cell Signaling Technology, Danvers, MA, USA). The membrane was washed again in PBS/0.5% Tween-20 and antigens were detected using the chemiluminescence substrate ChemiGlow® according to the manufacturer instructions (Alpha Innotech Corporation, San Leandro, CA, USA). Before probing for the total expression of PDGFr-β, the membrane was stripped 2 × 10 min in 200 mM glycine, 3.5 mM SDS, and 1% Tween-20 pH adjusted to 2.2 with HCl, washed 2 × 10 min in PBS, 1 × 5 min in 0.05 M Tris, pH 8.0, 0.138 M NaCl, 0.0027 M KCl, 0.5% Tween-20, followed by a 1-min methanol rinse and a final 5-min wash in 0.05 M Tris, pH 8.8, 0.138 M NaCl, 0.0027 M KCl, 0.5% Tween-20. Membrane was then re-probed with rabbit anti-human/anti-mouse PDGFr-β polyclonal Ab 3162 (Cell Signaling Technology, Danvers, MA) to determine total PDGFr-β. The subsequent gels were analyzed in the same manner to determine the expression of human PDGFr-β. Primary antibodies were mouse monoclonal anti-human PDGFr-β from Abcam (#ab10847) used at 1:55,000 dilution. ImmunoPure goat anti-mouse IgG (H + L) conjugated with horseradish peroxidase from Pierce (31430) was used as a secondary antibody at 1:300,000 dilution. Protein band intensity was measured using the ImageJ software [41] and normalized using the β-actin band intensity as the internal standard of the total protein load.

### 3.7. PDGF-B Immunoassays

Quantikine human and mouse PDGF-BB assays were purchased from R&D Systems, Inc. (Minneapolis, MN, USA). Human PDGF-BB assay uses the PDGFr-β/Fc chimera pre-coated onto a microplate to capture PDGF-BB. In the mouse PDGF-BB assay, anti-PDGF-BB MAb is pre-coated onto a microplate. R&D Systems report cross-reactivity with human PDGF-BB to be 38.1%. Both assays were performed according to the manufacturer instructions. The average total protein load per well was 0.7 mg. Reported concentrations of mouse PDGF-BB were adjusted for the cross-reactivity with human PDGF-BB.

### 3.8. Immunohistochemistry

Paraffin sections were rehydrated and the antigen retrieved using the standard protocol. The endogenous peroxidase activity was blocked with 3% H_2_O_2_ (5 min). After a gentle rinse in PBS, slides were washed for 5 min in PBS and blocked for 15 min with normal goat serum (KPL, Gaithersburg, MD, USA) for goat secondary antibodies or Serum Blocking Reagent G (R&D Systems, Inc., Minneapolis, MN, USA) for rabbit secondary antibodies. The slides were drained and the excess serum wiped off. The avidin blocking reagent (R&D) was applied for 15 min. Slides were gently rinsed in PBS and the biotin blocking reagent (R&D) was applied for 15 min. Slides were again gently rinsed in PBS. Slides were placed in a humidified chamber and the primary antibodies were applied. For the detection of PDGF-B, rabbit polyclonal IgG sc-127 (Santa Cruz Biotechnology, Inc.) at a concentration of 1 μg/mL was used. PDGFR-β was detected with either goat polyclonal IgG sc-1627 (Santa Cruz Biotechnology, Inc.) at a concentration of 1 μg/mL or rabbit polyclonal IgG 3162 (Cell Signaling Technology, Inc., Danvers, MA, USA) was used at a 1:50 dilution of the stock solution provided by vendor. After the overnight incubation, slides were washed 3 × 5 min in PBS. The appropriate biotinylated secondary antibodies (KPL) were applied for 60 min. Slides were washed 3 × 5 min in PBS and exposed to the peroxidase-labeled streptavidin (KPL) for 30 min. After a gentle rinse and 3 × 2 min washes with PBS, the DAD peroxidase substrate solution was applied for 20 min. Stained slides were rinsed and washed in distilled water (5 min) and counterstained with hematoxylin using standard protocols. Sections were photographed with a Nikon DXM 1200 digital camera mounted on a Nikon Optiphot microscope.

### 3.8. Statistical Analyses

In all summary statistics Student's t-test was used to compare the averages.

## 4. Conclusions

Data reported here suggest that in view of the massive activation of autocrine and paracrine PDGFr-β signaling pathways, some stroma-directed therapies may have minimal or no benefit in pancreatic cancer. Based on the compelling data presented here it is apparent that a scrupulous determination of PDGFr-β and PDGF-B status in *cancer* cells must be undertaken before any therapies targeting the stromal component of pancreatic cancer are contemplated.

## Supplementary Files

Supplementary File 1 Supplementary File (PDF, 823 KB)
