# Supplementary material for: Activation of PDGFr-β Signaling Pathway after Imatinib and Radioimmunotherapy Treatment in Experimental Pancreatic Cancer
Source: Cancers (Basel). 2011 May 25;3(2):2501–15. doi: 10.3390/cancers3022501 (PMC3757429; doi:10.3390/cancers3022501)
Supplement: Supplementary File 1 — Supplementary File (PDF, 823 KB) [file cancers-03-02501-s001.pdf]

Cancers **2011**, *3*, doi:10.3390/cancers3022501

OPEN ACCESS

cancers

ISSN 2072-6694
www.mdpi.com/journal/cancers

Supplementary

Activation of PDGFr-β Signaling Pathway after Imatinib and Radioimmunotherapy Treatment in Experimental Pancreatic Cancer

Michio Abe [1], Zbigniew P. Kortylewicz [2], Charles A. Enke [2], Elizabeth Mack [2] and Janina Baranowska-Kortylewicz [2,*]

[1] Minamata City Hospital and Medical Center, Minamata City, Kumamoto 867, Japan; E-Mail: PFG02651@nifty.ne.jp

[2] Department of Radiation Oncology, J. Bruce Henriksen Cancer Research Laboratories, University of Nebraska Medical Center, Omaha, NE 68198, USA; E-Mails: zkortylewicz@unmc.edu (Z.P.K.); cenke@unmc.edu (C.A.E.); elizabeth.mack@unmc.edu (E.M.)

* Author to whom correspondence should be addressed; E-Mail: jbaranow@unmc.edu; Tel.: +1-402-559-8906; Fax: +1-402-559-9127.

Figure S1. Sections of pancreatic human adenocarcinoma SW1990 xenografts grown subcutaneously in athymic mice given only PBS (controls, upper row) or treated with imatinib (lower row). Sections were cut from several xenografts to illustrate the tissue heterogeneity. The structure of these tumors and their heterogeneity is best viewed when the page size is at 500%. H&E, original magnification ×10.

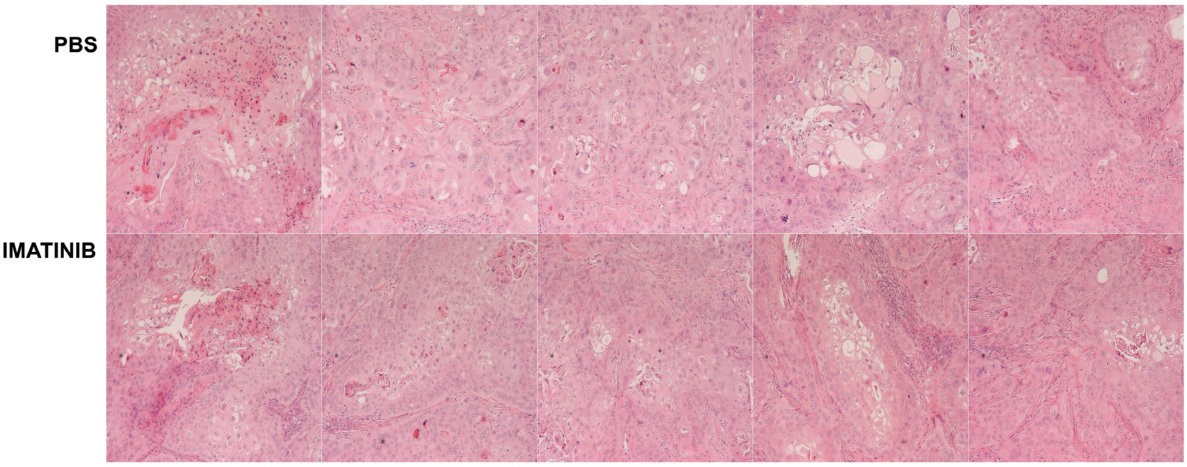

© 2011 by the authors; licensee MDPI, Basel, Switzerland. This article is an open access article distributed under the terms and conditions of the Creative Commons Attribution license (http://creativecommons.org/licenses/by/3.0/).
